# Development of a Mild Viral Expression System for Gain-Of-Function Study of Phytoplasma Effector *In Planta*

**DOI:** 10.1371/journal.pone.0130139

**Published:** 2015-06-15

**Authors:** Sin-Fen Hu, Yu-Hsin Huang, Chan-Pin Lin, Li-Yu Daisy Liu, Syuan-Fei Hong, Chiao-Yin Yang, Hsiao-Feng Lo, Ting-Yu Tseng, Wei-Yao Chen, Shih-Shun Lin

**Affiliations:** 1 Institute of Biotechnology, National Taiwan University, Taipei, Taiwan; 2 Genome and Systems Biology Degree Program, National Taiwan University, Taipei, Taiwan; 3 Agricultural Biotechnology Research Center, Academia Sinica, Taipei, Taiwan; 4 Departement of Plant Pathology and Microbiology, National Taiwan University, Taipei, Taiwan; 5 Department of Horticulture and Landscape Architecture, National Taiwan University, Taipei, Taiwan; 6 Department of Agronomy, National Taiwan University, Taipei, Taiwan; 7 Joint Center for Instruments and Researches, College of Bioresources and Agriculture, National Taiwan University, Taipei, Taiwan; Indiana University, UNITED STATES

## Abstract

PHYL1 and SAP54 are orthologs of pathogenic effectors of Aster yellow witches’-broom (AYWB) phytoplasma and Peanut witches’-broom (PnWB) phytoplasma, respectively. These effectors cause virescence and phyllody symptoms (hereafter leafy flower) in phytoplasma-infected plants. T_0_ lines of transgenic Arabidopsis expressing the *PHYL1* or *SAP54* genes (*PHYL1* or *SAP54* plants) show a leafy flower phenotype and result in seedless, suggesting that PHYL1 and SAP54 interfere with reproduction stage that restrict gain-of-function studies in the next generation of transgenic plants. *Turnip mosaic virus* (TuMV) mild strain (TuGK) has an Arg182Lys mutation in the helper-component proteinase (HC-Pro^R182K^) that blocks suppression of the miRNA pathway and prevents symptom development in TuGK-infected plants. We exploited TuGK as a viral vector for gain-of-function studies of *PHYL1* and *SAP54* in Arabidopsis plants. TuGK-PHYL1- and TuGK-SAP54-infected Arabidopsis plants produced identical leafy flower phenotypes and similar gene expression profiles as *PHYL1* and *SAP54* plants. In addition, the leafy flower formation rate was enhanced in TuGK-PHYL1- or TuGK-SAP54-infected Arabidopsis plants that compared with the T_0_ lines of *PHYL1* plants. These results provide more evidence and novel directions for further studying the mechanism of PHYL1/SAP54-mediated leafy flower development. In addition, the TuGK vector is a good alternative in transgenic plant approaches for rapid gene expression in gain-of-function studies.

## Introduction

Aster yellow witches’-broom (AYWB) phytoplasma causes virescence and phyllody symptoms in host plants [1]. Maclean et al. (2011) individually expressed several putative secreted AYWB phytoplasma proteins (SAPs) in Arabidopsis to identify the phytoplasma effector that induces these virescence and phyllody symptoms (hereafter leafy flower). Large-scale screening revealed SAP54 to be the effector causing leafy flower and crinkled silique phenotypes in Arabidopsis [2]. Furthermore, the PHYL1 effector of Onion yellows phytoplasma, an effector orthologous to SAP54, also results in a leafy flower phenotype in Arabidopsis expressing the *PHYL1* gene [3], suggesting that SAP54 and PHYL1 play roles in leafy flower formation. Our pervious study indicated that Peanut witches’-broom (PnWB) phytoplasma also occurs leafy flower symptoms in *Catharanthus roseus* plants [4], and its *PHYL1* gene has also been identified [5]. To evaluate the PHYL1 of PnWB function, the gain-of-function study in Arabidopsis plants is an approach to study the mechanism of leafy flower development. However, the transgenic Arabidopsis expressing *SAP54* (*SAP54* plants) have a seedless problem that restricted the further study in function of effectors. Therefore, an alternative strategy for *PHYL1*/*SAP54* gain-of-function *in vivo* that is independent from the transgenic approach is needed.

Since the 1990s, plant viral vectors have been developed to carry foreign genes for different purposes. Unlike the transgenic approach, a viral vector has several advantages, such as self-replication and long-distance movement, allowing the systemic spread of interesting foreign sequences for efficient expression or virus-induced gene silencing (VIGS) in plant [6–12]. A viral suppressor is a key regulator for counteracting the RNA-silencing defense in plants [13–15]. Because of silencing suppression, foreign proteins in viral vector-infected plants are expressed to a higher degree than in transgenic plants [16–18]. In addition, the viral suppressor interferes microRNA (miRNA) biogenesis, resulting in severe symptoms that affect the normal growth status of host plants [19, 20]. To overcome this issue, a deconstructed vector was applied for over-expression of a foreign gene through the expression of the required viral elements [21, 22]. Moreover, this deconstructed vector can also overcome the problems of size limitations and the deletion of foreign genes, which occurred with viral vectors [21].

*Tobacco rattle virus* (TRV) and *Potato virus X* (PVX), which has a weak suppressor in silencing suppression, have been widely applied for VIGS in loss-of-function studies [12]. A suitable viral vector, other than a VIGS vector, for rapid gain-of-function studies in infected wild-type or mutant plants is also important; especially when the transgene causes a lethal phenotype in seedlings of the next generation. Many reports have demonstrated that several virus species can express *green fluorescent protein* (*GFP*) gene for proving of the concept in viral vector development; however, functional study of gene is lacking [6, 23].

The attenuated *Turnip mosaic virus* (TuMV) strain (TuGK) has an Arg182Lys mutation in helper component-proteinase (HC-Pro^R182K^). HC-Pro^R182K^ is a mutant of a gene-silencing suppressor that has lost its ability to suppress the miRNA pathway, results in no symptoms in TuGK-infected *Arabidopsis thaliana* (hereafter Arabidopsis) and *Nicotiana benthamiana* plants [19, 24]. TuGK has been modified as a viral vector to express *GFP* for indicating and monitoring the virus location in the host [19]. An infectious, full-length cDNA clone of TuGK has been constructed in the mini binary vector pBD003 to generate pBD-TuGK, which can be directly inoculated into host plants via agro-infiltration to induce an initial infection [24]. TuGK-infected Arabidopsis plants is symptomless [19], making TuGK suitable for the over-expression of foreign genes in Arabidopsis plants, with few or no side effects due to the severe pathogenicity of the virus.

In this study, TuGK was used as a viral vector for rapid expression to study the function of the *PHYL1* and *SAP54* gene in Arabidopsis plants. Recombinant TuGK viruses could successfully express effectors in Arabidopsis and mimic the leafy flower phenotype of the transgenic plants, demonstrating the use of TuGK as a rapid system for gain-of-function studies *in planta*.

## Materials and Methods

### Plant materials and growth conditions

Arabidopsis seeds were surface sterilized and chilled at 4°C for 2 days before being sown on Murashige and Skoog (MS) medium with/without suitable antibiotics for selection. One-week-old seedlings that had germinated on the MS plates were transferred to soil. *N*. *benthamiana* seeds were sown in soil. All of the seedlings and plants were maintained in either a growth chamber or greenhouse (16 hr light/8 hr dark, 20 to 25°C).

### Construction of effector genes in the TuGK viral vector and virus infection

The *PHYL1* gene was amplified from cDNA extracted from PnWB-infected *C*. *roseus* plants by reverse transcription-polymerase chain reaction (RT-PCR) using the primers PNheI-SAP54 (PnWB) (5'-CAAGGCTAGCATGGATCCAAAACTTCCAGAA-3') and MSAP54-NheI (PnWB) (5'-CACAGCTAGCGTTTTTTTCATCATTTAAATC-3'), which contains *NheI* sites (underlined). The gene was then inserted into the pGEM-T easy vector (Promega) to generate pGEM-PHYL1. The *SAP54* gene of AYWB was amplified from pDONR207-SAP54 (provided by Dr. Saskia Hogenhout) using the primers PSAP54-NheI (5'-GTACAAGGCTAGCATGGATAAAGATATTGCAAGCACT-3') and MSAP54-NheI (5'-AAACACAGCTAGCATTATTTTCATCATTTAA-3'), which contains *NheI* sites (underlined). The *SAP54* and *PHYL1* genes were inserted into the pBD-TuGK viral vector [24] via *Nhe*I digestion and ligation to generate pBD-TuGK-SAP54 and pBD-TuGK-PHYL1.

The infectious viral clones were transformed into the *Agrobacterium tumefaciens* C58C1 strain, and virus infection was performed in *N*. *benthamiana* plants using the agro-infiltration procedure [24]. Four days after infiltration, the infiltrated leaves of *N*. *benthamiana* plants were collected to analyze viral infectivity and were used as the primary inoculum to mechanically inoculate Arabidopsis plants. Recombinant TuGK infection was performed in 2.5-week-old Col-0 or *dcl2-4*/*dcl4-1* double-mutant (*dcl2/4*) plants. Flower tissues from plants at 20 days post-inoculation (dpi) were used for gene profile evaluation.

### Longitudinal sectioning of shoot apical meristem and confocal microscopy

The shoot apical meristem (SAM) of 4 dpi TuGK- or TuGK-PHYL1-infected Arabidopsis and *N*. *benthamiana* plants were embedded in 5% agar and sectioned at 200-μm thickness using a microslicer (DTK-1000; Dosaka EM, Japan). The GFP fluorescence in the SAM was monitored using a Leica TCS SP5 II confocal laser-scanning microscope (Joint Center for Instruments and Researches, College of Bioresources and Agriculture, National Taiwan University) that was equipped with a multiline argon laser with a filter set for GFP fluorescence [excitation filter Acousto-optic Tunable filter 488, emission bandwidth 502 to 572 nm, PMT2 offset (-1.0)/grain (895.3)] and a filter set for chlorophyll fluorescence [excitation filter Acousto-optic Tunable filter 488, emission bandwidth 608 to 677 nm, PMT3 offset (0.0)/gain (855)]. All images were graphically arranged using Adobe Photoshop CS3 software (Adobe Systems Inc., Mountain View, CA, U.S.A).

### Transgenic Arabidopsis expressing *PHYL1*

The *PHYL1* gene was amplified from pGEM-PHYL1 with the primers FG-PHYL1-Pn (5'-CACCATGGATCCAAAACTTCCAGAAACTAGTAGCAG-3') and RG-PHYL1-Pn (5'-TTAGTTTTTTTCATCATTTAAATCATTTAA-3'). The PCR fragment was cloned into the pENTR/D-TOPO vector (Invitrogen) according to the manufacturer’s instructions to generate pENTR-PHYL1. The Gateway system was used to transfer the *PHYL1* gene to the pBA-DC-myc binary vector [25], generating pBA-PHYL1. The binary plasmid was transformed into the *Agrobacterium tumefaciens* ABI strain for transgenic Arabidopsis transformation using the floral-dip procedure [26]. The transformant lines were screened on MS medium containing 10 μg/ml Basta (Sigma).

### PHYL1 and SAP54 antiserum production

To express recombinant PHYL1 protein in *E*. *coli*, the gene was amplified from pGEM-PHYL1 using the primers PPn-NheI (5'-TATGGCTAGCATGGATCCAAAACTTCCAGA-3') and MPn-XhoI (5'-GGTGCTCGAGGTTTTTTTCATCATTTAAAT-3'), which contain *Nhe*I and *Xho*I sites (underlined). To express the recombinant SAP54 protein in *E*. *coli*, the gene was amplified from pDONR207-SAP54 using the primers PAY-NheI (5'-TATGGCTAGCATGGATAAAGATATTGCAAG-3') and MAY-XhoI (5'-GGTGCTCGAGATTATTTTCATCATTTAAAG-3'), which contain *Nhe*I and *Xho*I sites (underlined). The PCR fragments were digested with *Nhe*I/*Xho*I and ligated into pET28a (Novagen) that had been digested with the same restriction enzymes to generate pET-PHYL1 and pET-SAP54.

*E*. *coli* cells transformed with either pET-PHYL1 or pET-SAP54 were individually cultured in 500 ml of LB medium at 37°C until the absorbance at 600 nm reached 0.5. After that, the 0.5 mM isopropyl-D-1-thiogalactopyranoside (IPTG) was added in the culture and grown at 20°C for 6 hr. Recombinant protein purification and antiserum production were performed using modified protocols published by Chiu et al [27]. Approximately 1 mg of either recombinant PHYL1 or SAP54 protein was mixed with Freund’s adjuvant (1:1 v/v) and injected into a New Zealand white rabbit once per week; the procedure was repeated 4 times. For the first injection, the “complete” adjuvant was used, and the “incomplete” adjuvant was for the subsequent injections. The titer of the antiserum was analyzed using western blotting. Institutional Animal Care and Use Committee (IACUC) of National Taiwan University (NTU) approved the antiserum production in this study. The feeding and care of the experimental rabbits were bred at animal room of Institute of Biotechnology of NTU. All antiserum processing, including animal welfare, ameliorates suffering, and sacrifice, were performed according to institutional guidelines under the regulation of the IACUC of NTU and to laws of Taiwan on animal protection.

### Western blot analysis

Plant tissue was ground in 5–10 volumes (w/v) of 20 mM phosphate buffer, pH 7.0, and the plant extracts were suspended in 2 volumes (w/v) of 2× protein sample dye (2% SDS, 10% glycerol, 1% ß-mercaptoethanol, 0.005% bromophenol blue, and 50 mM Tris-HCl, pH 6.8), denatured 100°C for 10 min and then cooled on ice for 2 min. The protein samples were separated on 12% polyacrylamide-SDS gels and then transferred to a PVDF membrane (GE Healthcare) with transfer buffer (50 mM Tris-HCl, 40 mM glycine, 1 mM SDS, and 20% methanol). For TuMV coat protein (CP) detection, the CP antiserum was used at a 10,000× dilution (Chiu *et al*., 2013). For phytoplasma effector detection, SAP54 or PHYL1 antisera were used at a 10,000× dilution. For GFP detection, a GFP monoclonal antibody (GE Healthcare) was used at an 8000× dilution. HRP-conjugated anti-rabbit or anti-mouse antibodies (GE Healthcare) were used as secondary antibodies at a 10,000× dilution, and the signals were detected using WesternBright ECL (Advansta). The membrane was stained with staining solution (0.6 mM Coomassie blue R-250, 50% methanol, 10% acetic acid) to perform riblose-1,5-bisphosphate carboxylase (RUBISCO) staining as a loading control.

### Identification of flower-related gene expression in *SAP54* plants

Total RNA was extracted from normal flowers of Col-0 plants or leafy flowers of *SAP54* plants using TRIzol reagent (Invitrogen) according to the manufacturer’s protocol. One sample for each condition was used for the NGS data. The whole-transcriptome profiles of the Col-0 and *SAP54* plants were analyzed using an Illumina Hiseq 2000 (Genomics BioSci & Tech Co.) The raw transcriptome reads of Col-0 and *SAP54* plants are available in the NCBI Short Read Archive under accession number SRR1979100 (normal flower of Col-0 plant), and SRR1979105 (leafy flower of *SAP54* plant). The flower-related gene expression profiles were identified from the whole-transcriptome profiles. The raw reads for the *AtAP3* (AT3G54340), *AtSVP* (AT2G22540), *AtFT* (AT1G65480), and *AtUBQ10* (AT4G05320) genes from Col-0 and *SAP54* flower tissues were used to calculate the reads per kilobase per million mapped reads (RPKM) values of the 3 flower-related genes using the CLC Genomics Workbench 7.5.1 (CLC bio).

### Real-time RT-PCR

Total RNA was extracted from 0.1 g of plant tissue using the Trizol reagent (Invitrogen). RT was performed using the SuperScript III first-strand synthesis system (Invitrogen) following the manufacturer’s instructions. Real-time PCR was performed using a LightCycler 480 instrument (Roche) with four sets of primers: PAP3 (5'-GAGTGTTTGGACGAGCTTGA-3') and MAP3 (5'-TTCTTGGTGGTCTCGATCTG-3') for the amplification of the *AtAP3* gene; PSVP (5'-GGAATGCAATTGATGGATGA-3') and MSVP (5'-TCCTTCCTCGTACACAGCAG-3') for the amplification of the *AtSVP* gene; PFT (5'- CCTTTGGCAATGAGATTGTG-3') and MFT (5'-GCCAAGCTGTCGAAACAATA-3') for the amplification of the *AtFT* gene; and PUBQ10 (5'-CACTTCACTTGGTCTTGCGT-3') and MUBQ10 (5'-TATCCTGGATCTTGGCCTTC-3') for the amplification of the *AtUBQ10* gene. The *AtAP3*, *AtSVP*, *AtFT*, and *AtUBQ10* transcripts were quantified via a relative cycle threshold (Ct) method. All experiments were performed in triplicate to compensate for possible loading errors. The relative expression levels were calculated based on the ΔΔCt value, and each sample was normalized according to the expression levels of *AtUBQ10*.

## Results

### TuGK expresses phytoplasma effectors *in planta*

**Fig 1:**
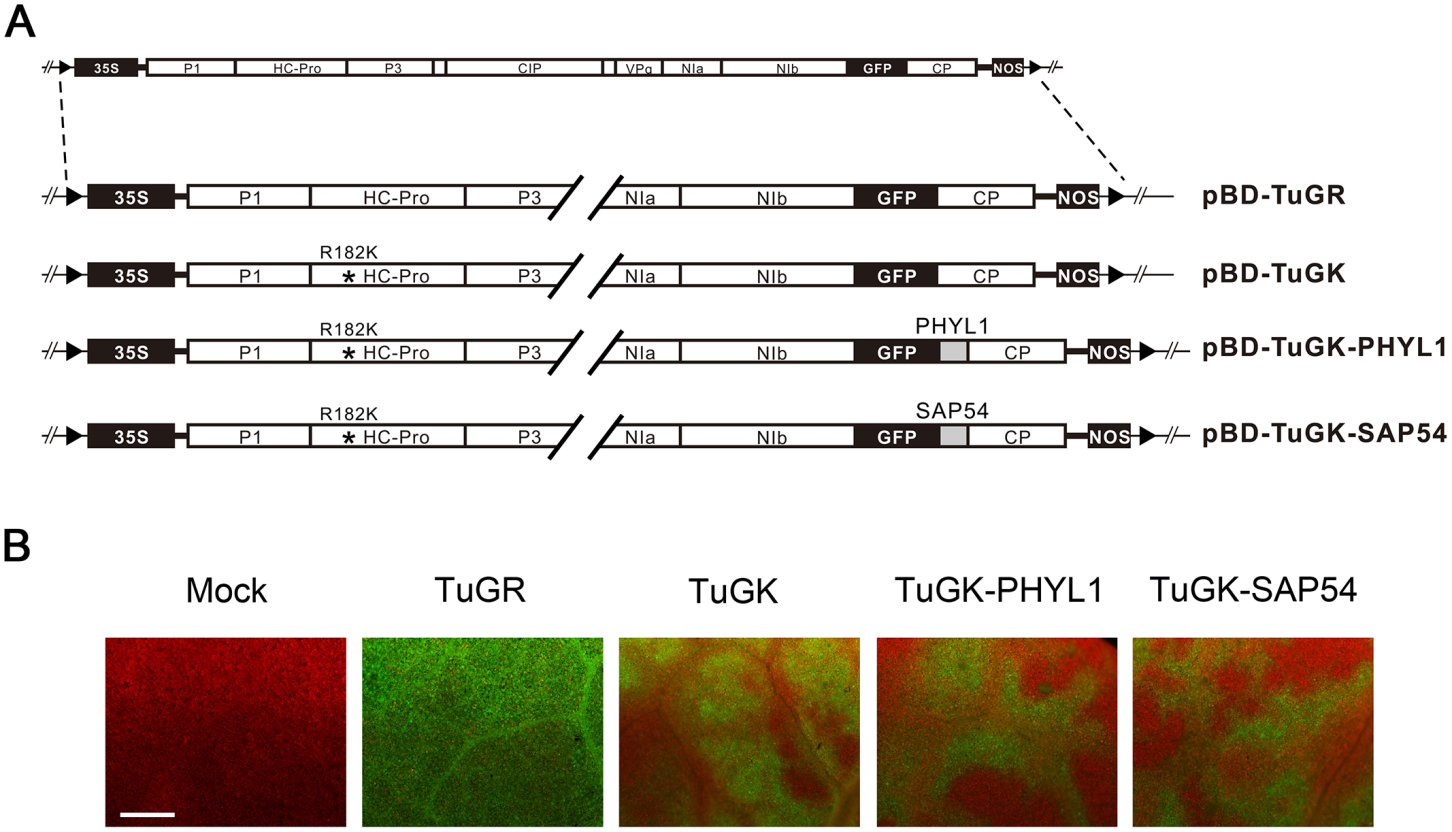
Recombinant TuGK viral vectors expressing phytoplasma effector genes in *Nicotiana benthamiana* plants. A, Schematic diagram of pBD-TuGR, pBD-TuGK, pBD-TuGK-PHYL1, and pBD-TuGK-SAP54. The TuMV genes are indicated by white boxes. The *green fluorescent protein* gene (*GFP*) is indicated by a black box. The *SAP54* of AYWB and *PHYL1* of PnWB genes are indicated by gray boxes. Asterisks (*) indicate the Arg182Lys mutation (R182K) in HC-Pro. The *35S* promoter and *NOS* terminator are indicated by black boxes. The left border (LB) and right border (RB) sequences are indicated. B, Viral infectivity assay of the recombinant viruses. GFP expression by TuGR, TuGK, TuGK-PHYL1, and TuGK-SAP54 in *N*. *benthamiana* plants. Images were acquired of *N*. *benthamiana* plants at 4 dpi with fluorescence microscopy at 400× magnification. Bar, 25 μm.

pBD-TuGK was used to carry the *PHYL1* or *SAP54* gene (Fig 1A). Both effector genes were fused at the C-terminus of GFP and inserted between the *NIb* and *CP* genes with NIa protease cleavage sites (Fig 1A). pBD-TuGK-PHYL1 and pBD-TuGK-SAP54 were produced by the TuGK-PHYL1 and TuGK-SAP54 recombinant viruses in *N*. *benthamiana* plants after agro-infiltration and exhibited a GFP signal under fluorescence microscopy (Fig 1B). Compared with plants infected with a wild-type TuMV that expresses GFP (TuGR), GFP fluorescence was lower in the TuGK-, TuGK-PHYL1-, and TuGK-SAP54-infected plants (Fig 1B). Thus, TuGK can express both effectors at lower expression levels.

### Evaluating the efficiency of PHYL1 and SAP54 expression by TuGK

**Fig 2:**
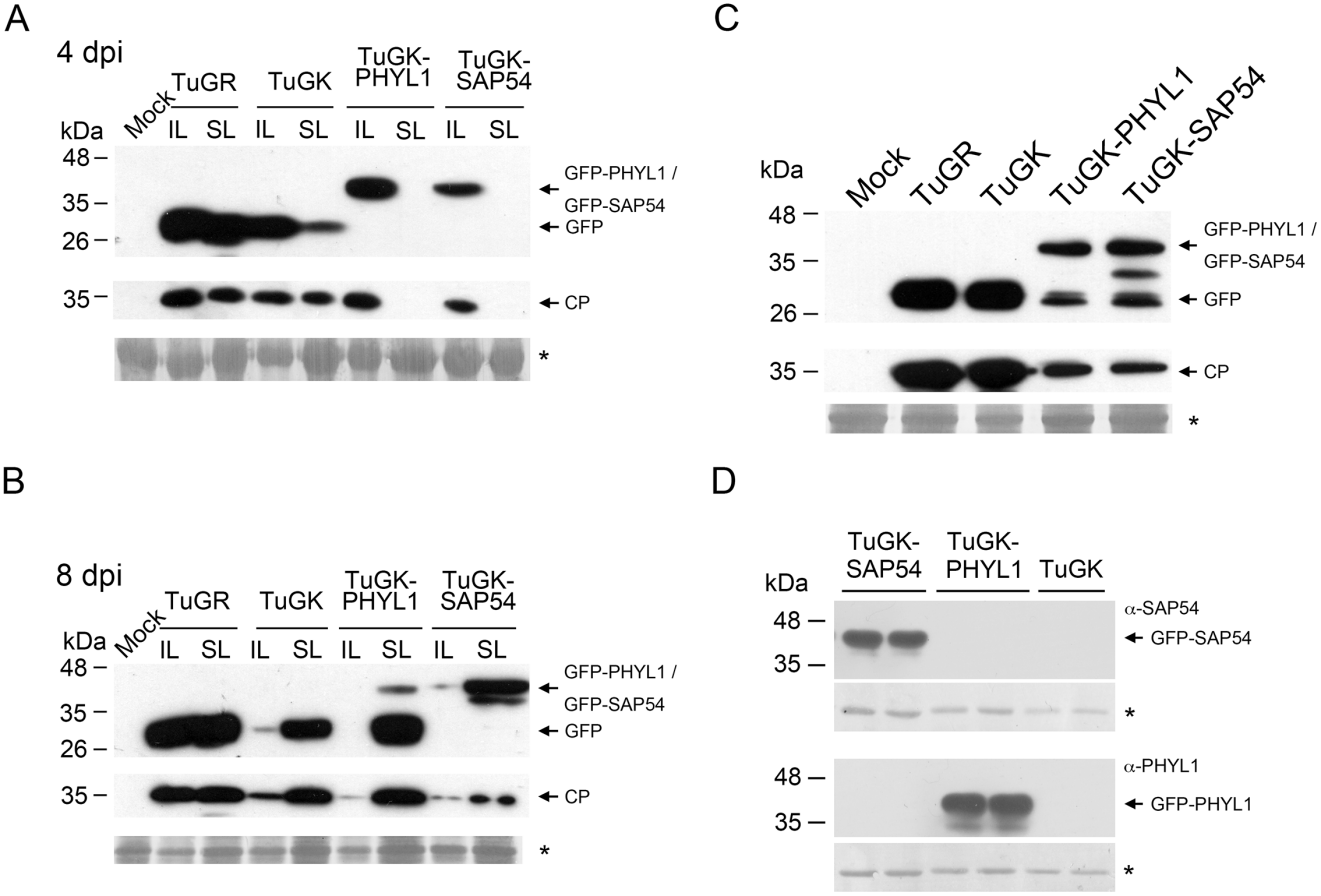
Detection of effector expression in TuGK-PHYL1- and TuGK-SAP54-infected plants. Foreign gene expression in TuMV-infected *Nicotiana benthamiana* plants at 4 dpi (A) or 8 dpi (B). IL indicates an inoculated leaf, and SL indicates a systemic leaf. C, Foreign gene expression in TuMV-infected Arabidopsis *dcl2-4*/*dcl4-1* double-mutants (*dcl2/4*). TuGR, a wild-type TuMV that expresses the *green fluorescent protein* (*GFP*) gene; TuGK, an HC-Pro Arg182Lys mutant of TuMV that expresses the *GFP* gene; TuGK-PHYL1, TuGK expressing the *GFP*-*PHYL1* fusion gene; TuGK-SAP54, TuGK expressing the *GFP*-*SAP54* fusion gene. The upper panels were detected using an 8,000× dilution of the GFP antibody. The lower panels were detected using a 10,000× dilution of TuMV coat protein (CP) antiserum. D, SAP54 and PHYL1 detection using specific antisera at 10,000× dilutions. The large subunit of ribulose-1,5-bisphosphate carboxylase (*) was used as a loading control.

TuGR and TuGK expressed GFP in both inoculated leaves (ILs) and systemic leaves (SLs) of *N*. *benthamiana* plants at 4 dpi (Fig 2A). In contrast, only ILs showed TuGK-PHYL1 and TuGK-SAP54 infection at 4 dpi (Fig 2A), suggesting that phytoplasma effectors might interfere with TuGK replication and infection in host plants. Surprisingly, the *PHYL1* gene was deleted from the TuGK genome in SLs, leaving only GFP (25 kDa) and truncated fusion proteins in SLs at 8 dpi (Fig 2B). Note, the *SAP54* gene was also deleted from TuGK in some instances (data not shown).

TuGK-infected leaves of *N*. *benthamiana* plants at 4 dpi were used as the inoculum to inoculate Arabidopsis plants. TuGK-PHYL1- and TuGK-SAP54-infected Col-0 plants showed partial deletion of the GFP-PHYL1 and GFP-SAP54 fusion proteins at 20 dpi; nevertheless, high levels of the fusion proteins persisted (Fig 2C). Moreover, SAP54 and PHYL1 antisera specifically detected GFP-SAP54 and GFP-PHYL1, respectively, in the infected Arabidopsis plants (Fig 2D), suggesting that TuGK expresses either the *PHYL1* or *SAP54* effector gene, respectively. Although PHYL1 and SAP54 exhibit 60.4% similarity, no cross-reactivity was observed by western blotting, demonstrating the high specificity of the antisera in distinguishing these two effectors.

### TuGK was detected in SAM

**Fig 3:**
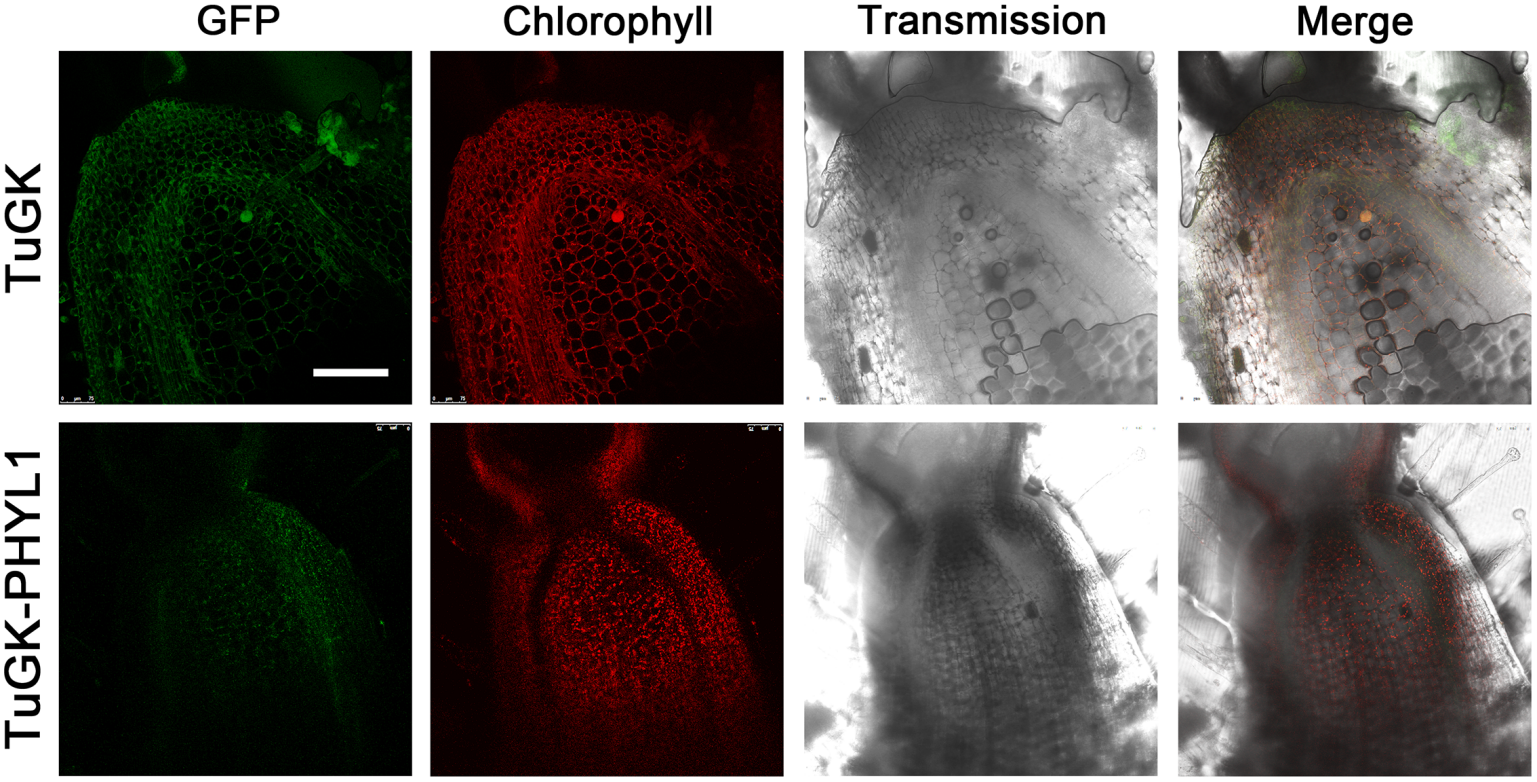
Longitudinal section of the shoot apical meristem (SAM) of *Nicotiana benthamiana* plants. The TuGK- and TuGK-PHYL1-infected SAM sections were evaluated by confocal microscopy. Complete stacks demonstrate that GFP (green), GFP-PHYL1 (green) and chlorophyll (red) were present in the SAM region. The SAM tissues were corrected at 4 dpi of TuGK- or TuGK-PHYL1-infected plants. Bar, 100 μm.

The SAM is an important position in plants with regard to leafy or flower organ determination. We assume that 35S promoter-driven effectors affect the gene expression in the SAM of *PHYL1* and *SAP54* plants, resulting in leafy flowers. Next, we evaluated whether TuGK can migrate to the SAM and express the effector in the meristem. The results of longitudinal section showed GFP fluorescence in the SAM regions of TuGK- and TuGK-PHYL1-infected *N*. *benthamiana* plants (Fig 3). Moreover, the SAM of Arabidopsis also showed GFP fluorescence of TuGK (S1 Fig). These results indicated that TuGK and TuGK-PHYL1 migrate to the meristem and express GFP-PHYL1 in the SAM region. Therefore, TuGK-delivered PHYL1 might affect gene expressions in the meristem as in *PHYL1* plants.

### Recombinant TuGK-PHYL1 and TuGK-SAP54 trigger leafy flower phenotypes in infected Arabidopsis

**Fig 4:**
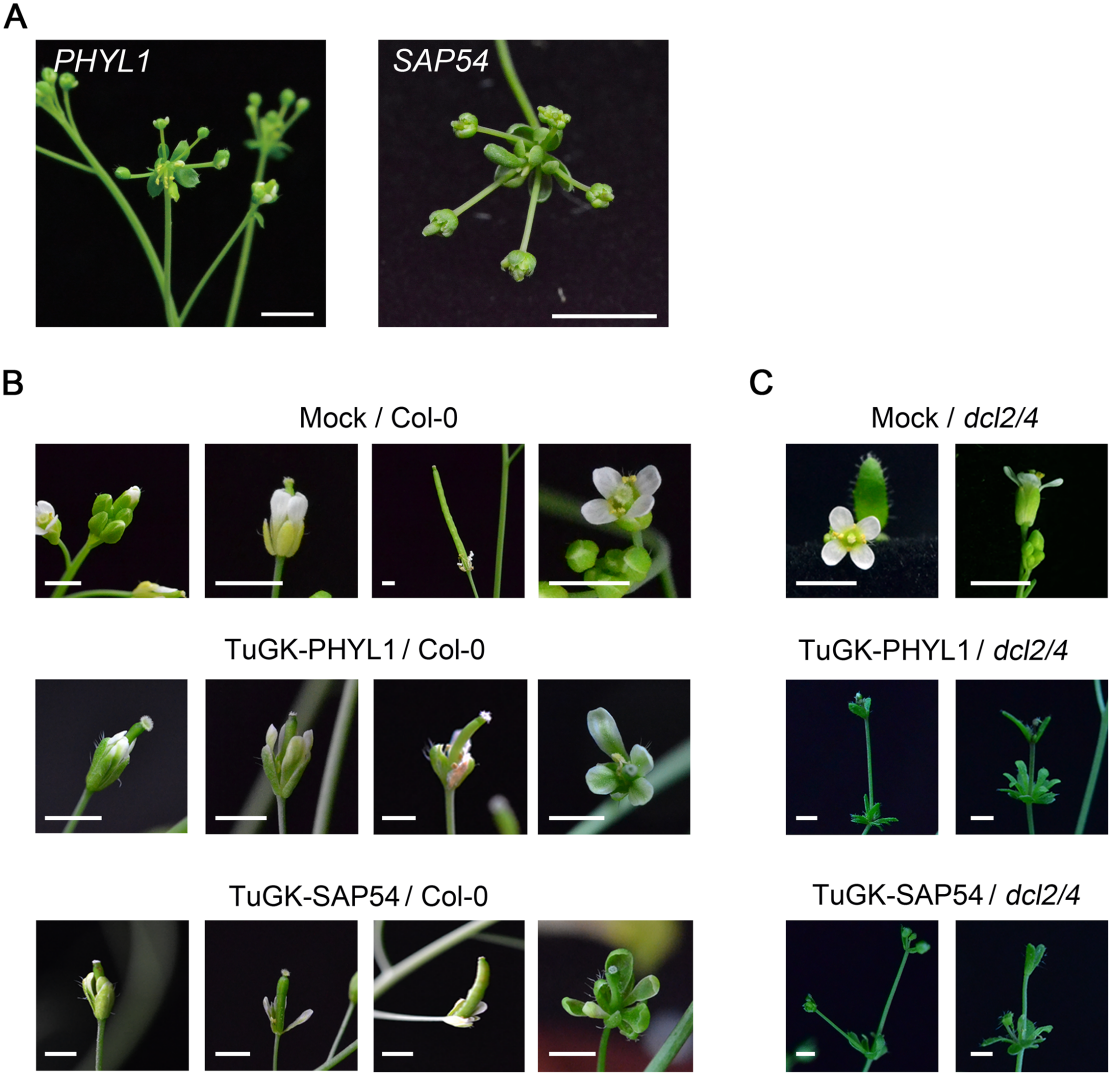
Leafy flower phenotypes of TuGK-PHYL1- and TuGK-SAP54-infected Arabidopsis plants. A, The leafy flower phenotypes of *SAP54* and *PHYL1* plants. Bar, 0.2 cm. The leafy flower phenotypes of TuGK-SAP54- or TuGK-PHYL1-infected Col-0 (B) and *dcl2/4* (C) plants. Mock indicates inoculation with buffer alone. The photographs were taken at 20 dpi. Bar, 0.2 cm.

*SAP54* plants (kindly provided by Dr. Hogenhout) display a leafy flower phenotype (Fig 4A) [2], indicating that the SAP54 effector plays an important role in controlling phyllody symptoms in phytoplasma-infected plants. Moreover, our data showed a leafy flower phenotype for transgenic Arabidopsis expressing *PHYL1* (Fig 4A), suggesting that both effectors exert an identical function. To test whether TuHG-PHYL1 and TuGK-SAP54 can trigger the leafy flower phenotype, both recombinant viruses were infected into Arabidopsis plants. At 20 dpi, leafy flowers were observed in the TuGK-PHYL1- and TuGK-SAP54-infected Col-0 plants, whereas the mock-infected Col-0 plants showed normal flowers (Fig 4B). In addition, the *dcl2/4* plant is a double mutant in *Dicer-like 2* (*DCL2*) and *Dicer-like 4* (*DCL4*) genes, which is more susceptible to TuGK infection [19]. The TuGK-PHYL1- and TuGK-SAP54-infected *dcl2/4* plants also showed leafy flowers (Fig 4C). Neither TuGK-PHYL1 nor TuGK-SAP54 resulted in severe symptoms in Col-0 or *dcl2/4* plants (Fig 4B and 4C). These results suggested that TuGK delivers these effectors to SAM and trigger leafy flower.

### The TuGK vector enhances the phenotype formation rate

In *PHYL1* plants, the *PHYL1* gene was constructed under the control of the 35S promoter in the pBA-DC-myc binary vector and was transferred into Arabidopsis for over-expression. The T0 lines of the *PHYL1* plants showed a low ratio of leafy flower formation. Of the 774 individual T0 seedlings of *PHYL1* plants, which all showed Basta resistance (a selection marker), only 46 showed a leafy flower phenotype (Table 1). In this case, the leafy flower formation rate is 5.9% in this transgenic approach (Table 1). A similar phenomenon was also observed in the T0 seedlings of *SAP54* plants (data not shown), suggesting the existence of unclear mechanism(s) influence the effector-mediated leafy flower formation.

**Table 1:** Comparison of the leafy flower rate between transgenic plants and recombinant TuGK-infected plants.

	Phenotype				
	Leaf flower^a^	Normal flower^b^	Total plants Prop.^c^	(%)	*p*-value^d^
*PHYL1* plant^e^	46	728	774	5.9	NA
TuGK-PHYL1^f^/Col-0	11	48	59	18.6	2×E-4
TuGK-PHYL1/*dcl2/4*	15	44	59	25.4	3.1×E-8
TuGK-SAP54/Col-0	5	10	15	33.3	1.9×E-5
TuGK-SAP54 /*dcl2/4*	2	11	13	15.4	0.16

^a^The number of plants showing the leafy flower phenotype.

^b^The number of plants showing the normal flower phenotype.

^c^The proportions of the leafy-flower plants.

^d^The *p*-value of Chi-squared test.

^e^Transgenic Arabidopsis expressing the *PHYL1* gene.

^f^Arabidopsis plants infected with TuGK-PHYL1 or TuGK-SAP54.

The leafy flower rates were 18.6% and 25.4% in TuGK-PHYL1-infected Col-0 and *dcl2/4* plants, respectively, 3 to 5 times the ratio compared with the transgenic approach (Table 1). Similarly, leafy flower rates of 15.4 to 33.3% were found for TuGK-SAP54-infected Arabidopsis (Table 1). These data indicated that the PHYL1- and SAP54-mediated leafy flower formation rate could be enhanced through the TuGK expression approach. Moreover, the DCL2/DCL4-dependent virus defense system might interfere with recombinant virus infection and result in a reduction in the expression of foreign genes [19]. Despite the results of TuGK-SAP54 infection, TuGK-PHYL1 triggered a leafy flower formation rate in *dcl2/4* plants of 25.4%, whereas a formation rate of 18.6% was observed for TuGK-PHYL1-infected Col-0 plants (Table 1), suggesting that TuGK-expressed PHYL1 in *dcl2/4* plants can also enhance the phenotype formation.

### Flower-related gene expression profiles in TuGK-PHYL1- and TuGK-SAP54-infected leafy flowers

**Fig 5:**
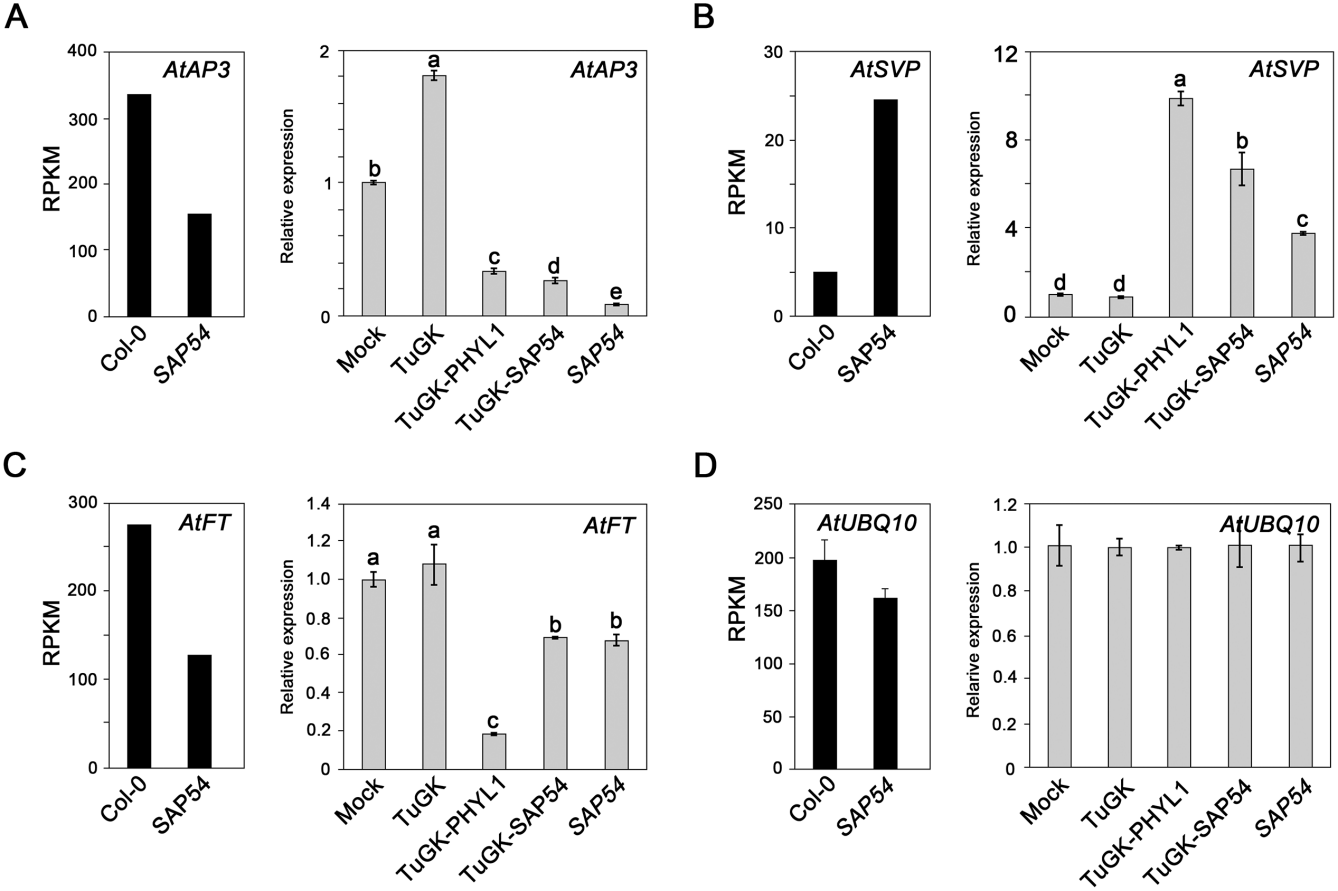
Flower-related gene expression in TuGK-PHYL1-and TuGK-SAP54-infected Arabidopsis flower tissues. The expression profiles of the *AtAP3* (A, left panel), *AtSVP* (B, left panel), *AtFT* (C, left panel), and *AtUBQ10* (D, left panel) genes in flower-tissues of Col-0 and *SAP54* plants were evaluated by transcriptome deep sequencing. The reads per kilobase per million mapped reads (RPKM) values are used to indicate gene expression levels. The bars represent the SE of 6 *AtUBQ10* isoforms (*n* = 6). Real-time RT-PCR evaluation of *AtAP3* (A, right panel), *AtSVP* (B, right panel), *AtFT* (C, right panel), and *AtUBQ10* (D, right panel) expression in various recombinant TuGK virus-infected *dcl2-4*/*dcl4-1* (*dcl2/4*) plants at 20 dpi. *SAP54* represents *SAP54* plants. The bars represent the SE (*n* = 3). The relative expression levels were normalized to the *AtUBQ10* level. The letters indicate significant differences among these relative expression levels of the RNA samples tested by Fisher’s least significant difference (LSD) method at ‘alpha’ = 0.05, with false discovery rate (FDR) correction after an analysis of variance (ANOVA).

To compare the gene expression profiles of Col-0 and *SAP54* plants, we evaluated the expression levels of 3 flower-related genes (*AtAP3*, *AtSVP*, and *AtFT*) in flower tissues from Col-0 and *SAP54* plants based on their transcriptome profiles (Fig 5). The *AtAP3* and *AtFT* genes, which promote flowering, were down-regulated in *SAP54* plants, whereas the flowering repressor *AtSVP* was up-regulated (Fig 5).

Next, real-time PCR was used to evaluate *AtAP3*, *AtSVP*, and *AtFT* expression levels in TuGK-PHYL1- and TuGK-SAP54-infected Arabidopsis (Fig 5). *AtUBQ10* is a housekeeping gene that has been used as a references gene for real-time RT-PCR normalization in many studies [28–30]. *AtUBQ10* has 6 alternative splicing isoforms (S1 Table), and the transcriptome profile indicated no significant differences of the average RPKM of 6 isoforms (*p*-value of t-test is 0.314) between Col-0 and *SAP54* plants (Fig 5D, left panel). Moreover, the real-time RT-PCR primer set for *AtUBQ10* was designed based on the conserved region of the 6 isoforms, and the real-time RT-PCR results indicated that *AtUBQ10* expression levels are consistent in virus-infected or uninfected Arabidopsis plants (Fig 5D, right panel). These results indicated that *AtUBQ10* expression is stable under the various conditions and treatments in this study and that it can be used as a references gene for normalization of flower-related gene expression.

The expression levels of *AtAP3* and *AtFT* were repressed in TuGK-PHYL1-infected or TuGK-SAP54-infected Arabidopsis and *SAP54* plants compared to mock-infected plants, whereas *AtSVP* was up-regulated in these plants (Fig 5A, 5B and 5C; right panels). These real-time RT-PCR results are consistent with the deep sequencing results, indicating that the TuGK expression system is consistent with the transgenic approach.

## Discussion

### The lack of symptoms of TuGK-infected plants is helpful for phenotype observation

Foreign gene expression by viral vectors *in planta* has an advantage in shortening the development time compared with transgenic plants. However, most viral vectors are constructed from viral strains that cause severe symptoms and affect the normal growth status of the plant host. Thus, it can be difficult to distinguish whether the phenotypes are caused by the expressed gene or plant immune responses. A deconstructed vector approach utilizes the minimum required viral elements for efficient expression; the missing functions can be provided by non-viral components to avoid symptoms. The deconstructed approach thus provides a solution for the symptom issue; however, the vector delivery system still relies on the agrobacterium-mediated transgenic method.

The HC-Pro^R182K^ of TuGK is a mutant defective in miRNA pathway suppression, resulting in a lack of symptoms in infected plants [19]. Therefore, TuGK can be used to perform unambiguous gain-of-function analyses in infected plants. Currently, the high-throughput next-generation (NGS) technique provides a powerful method to analyze whole-transcriptome profiles. Hundreds to thousands of candidate genes can be identified via high-throughput network analysis [4], and an efficient way to verify the functions of these candidate genes is needed. The TRV vector with the Gateway recombinant system has been developed for efficiency in constructing VIGS sequences of interests [12]. Therefore, the Gateway cassette can be employed with the TuGK vector for future high-throughput gain-of-function analyses.

The stability of a heterologous gene in a viral vector is dependent on the whether the gene interferes with the viral infection pathway. In addition, losing the foreign gene is strongly depended on demographic conditions [31]. For instance, the *GFP* gene is more stable in the short-term passages of TuMV, whereas the losing *GFP* gene in the long-term passage [31]. We assume the PHYL1 and SAP54 effectors might cause side effect to interfere with TuMV infection, whereas the harmless *GFP* gene can be passed to many progenies by viral vectors [10].

### TuGK can deliver effectors to the meristem and trigger leafy flower development

It is generally considered that the meristem of a plant is a cell division-active and virus-free region [32]. However, recent studies have demonstrated that several viruses, including *Tobacco ringspot virus*, *Pepper ringspot virus*, PVX, *Odontoglossum ringspot virus*, and *Barley stripe mosaic virus*, can infect the meristem region [32–37]. The confocal section data indicated that TuMV migrates to the meristem, suggesting that a TuGK-delivered foreign gene can affect host gene regulation in the meristem, resulting in altered morphology. Indeed, TuGK-PHYL1- or TuGK-SAP54-infected Arabidopsis showed a leafy flower phenotype identical to *PHYL1* and *SAP54* plants.

Our pervious study demonstrated that PnWB-mediated leafy flowers in *C*. *roseus* plants, which show up-regulation of *CrSVP1* and *CrSVP2*, but down-regulation of *CrAP3*.*1*, *CrAP3*.*2*, and *CrFT* [4]. The gene expression profiles in TuGK-PHYL1- and TuGK-SAP54-infected Arabidopsis plants indicated that *AtAP1*, *AtSVP*, and *AtFT* expressions are consistent as transgenic Arabidopsis and PnWB-infected *C*. *roseus* plants. Therefore, these data indicated that the TuGK vector can be used for gain-of-function studies. Moreover, the T0 lines of *PHYL1* and *SAP54* plants showed severe leafy flowers that did not produce seeds. TuGK-PHYL1 and TuGK-SAP54 infection of Arabidopsis provides sufficient experimental marital and can infect various mutant plants for further genetic analysis.

Furthermore, based on these data, we can immediately ask whether the PHYL1 or SAP54 effector acts similarly to the HC-Pro in silencing suppression or compensate the loss-of-miRNA suppression of HC-Pro^R182K^. Different pathogen effectors have been shown to exhibit synergistic effects upon co-infection [38]. However, TuGK-PHYL1- and TuGK-SAP54-infected Arabidopsis plants did not exhibit the severe yellow mosaic symptoms of TuMV, indicating that PHYL1 and SAP54 did not compensate for the loss-of-function of HC-Pro^R182K^.

### The TuGK vector enhances the phenotype formation rate

The leafy flower formation rate of the T0 lines of *PHYL1* plants was 5.9% (Table 1) and a similar phenomenon was also observed for *SAP54* plants (data not shown). Most of Basta-resistant T0 lines of *PHYL1* or *SAP54* plants produced normal flowers. We assume that the protein stability or another unclear mechanism affects PHYL1- or SAP54-mediated leafy flower formation. However, the phenotype formation rate of the TuGK-PHYL1- or TuGK-SAP54-infected plants was increased by 2.6 to 5.6 times compared with the *PHYL1* or *SAP54* plants. These data indicated that the TuGK vector enhances the phenotype formation.

## Conclusions

In this study, we used the TuGK mild strain, which lacks the ability to suppress the miRNA pathway, as a vector for a gain-of-function study and found phenotypes and gene expression levels similar to those of transgenic plants. Moreover, the TuGK expression system serves as an additional strategy for effector expression to bypass the issue of lack of seeds with *PHYL1* and *SAP54* plants. Combination of the Gateway recombinant system with the TuGK vector will facilitate future cloning and efficient screening in gain-of-function studies.

## Supporting Information

S1 Fig Longitudinal section of the shoot apical meristem (SAM) of Arabidopsis plants. The mock- and TuGK-infected SAM sections were evaluated by confocal microscopy. Complete stacks demonstrate that GFP (green), and chlorophyll (red) were present in the SAM region. The SAM tissues (white dashed-line boxes) were corrected at 7 dpi of mock- or TuGK-infected Arabidopsis plants. Bar, 200 μm.
(TIF)

S1 Table The read counts and RPKM of 6 *AtUBQ10* isoforms in Col-0 and *SAP54* plants. (DOCX)
