# Supplementary figures and images for: Development of a Mild Viral Expression System for Gain-Of-Function Study of Phytoplasma Effector In Planta
Source: PLoS One. 2015 Jun 15;10(6):e0130139. doi: 10.1371/journal.pone.0130139 (PMC4468105; doi:10.1371/journal.pone.0130139)

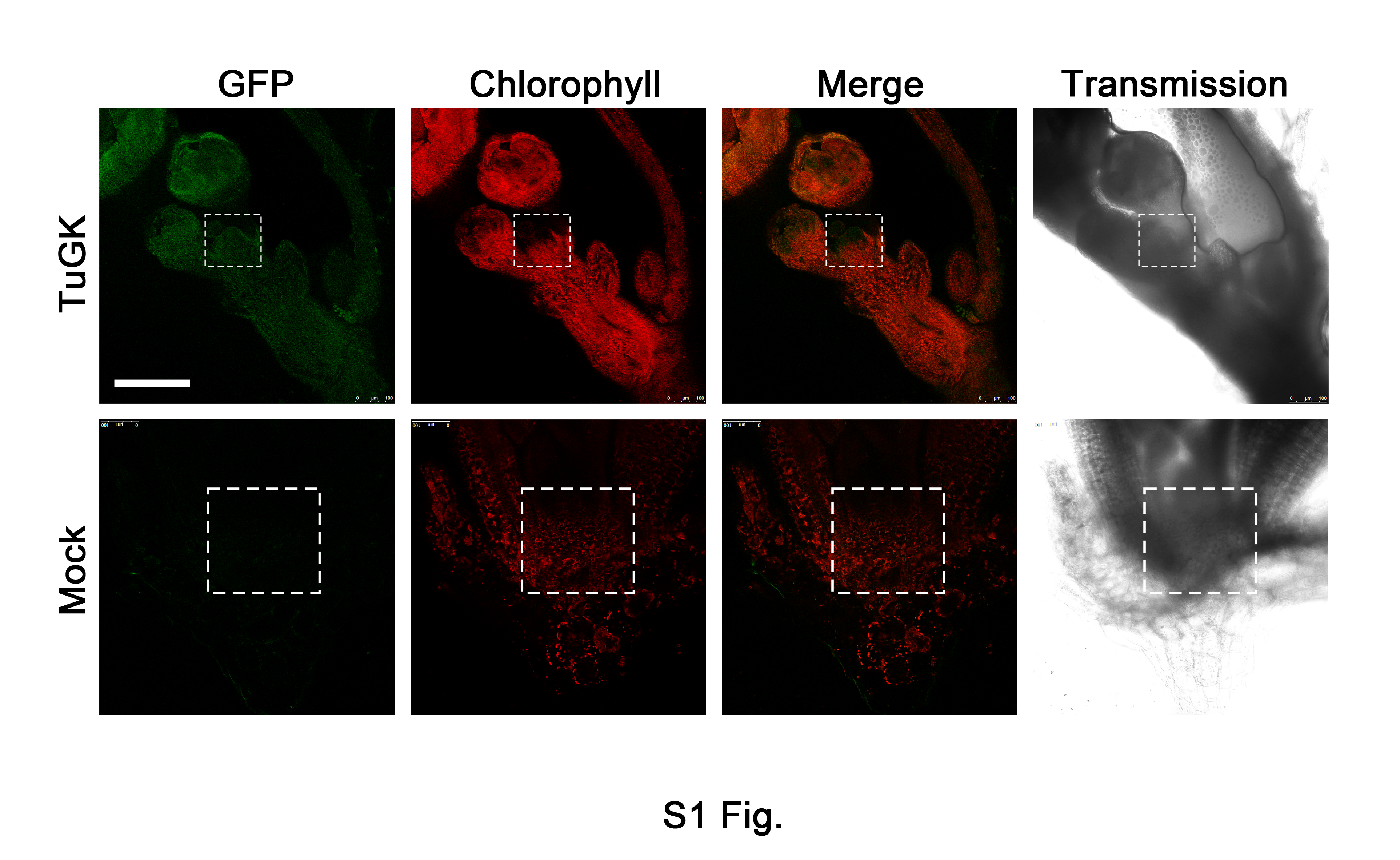

Supplement: S1 Fig — The mock- and TuGK-infected SAM sections were evaluated by confocal microscopy. Complete stacks demonstrate that GFP (green), and chlorophyll (red) were present in the SAM region. The SAM tissues (white dashed-line boxes) were corrected at 7 dpi of mock- or TuGK-infected Arabidopsis plants. Bar, 200 μm. (TIF) [file pone.0130139.s001.tif]
