# Supplementary material for: Development of a Mild Viral Expression System for Gain-Of-Function Study of Phytoplasma Effector In Planta
Source: PLoS One. 2015 Jun 15;10(6):e0130139. doi: 10.1371/journal.pone.0130139 (PMC4468105; doi:10.1371/journal.pone.0130139)
Supplement: S1 Table — (DOCX) [file pone.0130139.s002.docx]

**S1 Table. The read counts and RPKM of 6 *AtUBQ10* isoforms in Col-0 and *SPA54* plants.**

| AGI | Transcript length (bp) | Reads^a^ | | RPKM^b^ | |
| --- | --- | --- | --- | --- | --- |
|  |  | Col-0^c^ | *SAP54*^d^ | Col-0 | *SAP54* |
| AT4G05320.1^e^ | 1,432 | 3,830 | 4,822 | 107.61 | 134.12 |
| AT4G05320.2 | 1,835 | 6,571 | 4,746 | 144.07 | 103.02 |
| AT4G05320.3 | 1,550 | 3,642 | 7,118 | 94.54 | 182.92 |
| AT4G05320.4 | 1,625 | 14,637 | 9,874 | 362.40 | 242.03 |
| AT4G05320.5 | 1,204 | 5,352 | 3,238 | 178.85 | 107.12 |
| AT4G05320.6 | 1,270 | 9,267 | 6,138 | 293.58 | 192.51 |

^a^The read amounts of transcriptome that was analyzed by next-generation sequencing (NGS).

^b^Reads per kilobase per million mapped reads.

^c^The non-transgenic *Arabidopsis thaliana* Columbia strain. The total transcriptomic read counts of flower tissues from Col-0 plants are 12,422,826 reads.

^d^The transgenic Arabidopsis expressing *SAP54* gene. The total transcriptomic read counts of flower tissues from *SAP54* plants are 12,552,967 reads.

^e^The AGI numbers of *AtUBQ10* isoforms.
